# Healthcare beliefs, health information seeking, and healthcare setting preferences among women who inject drugs by community supervision status

**DOI:** 10.1186/s40352-021-00135-9

**Published:** 2021-04-16

**Authors:** Ariel Hoadley, Sarah Bauerle Bass, Jesse Brujaha, Paul A. D’Avanzo, Patrick J. Kelly

**Affiliations:** 1 Department of Social and Behavioral Sciences, Temple University College of Public Health, 1301 Cecil B. Moore Ave., Ninth Floor, Philadelphia, PA 19122 USA; 2 Risk Communication Laboratory, Temple University College of Public Health, 1301 Cecil B. Moore Ave., Ninth Floor, Philadelphia, PA 19122 USA

**Keywords:** Community supervision, Patient preference, Health information seeking, Injection drug use, Healthcare setting

## Abstract

**Objective:**

Women on community supervision who inject drugs have significant unmet healthcare needs. However, it remains unclear how the intersection of community supervision and injection drug use influences healthcare experiences and service setting preferences. The present study examines whether the intersection of community supervision and injection drug use is associated with differences in women’s healthcare beliefs, healthcare experiences, and service setting preferences.

**Methods:**

A secondary analysis was conducted on a previously collected sample of women who inject drugs recruited from a syringe exchange and social service organization for a cross-sectional survey. Participants (*N* = 64) were mostly White (75%), and more than a quarter were currently on probation or parole (26%).

**Results:**

Independent samples *t*-tests and chi-square tests revealed no significant differences on sociodemographic variables by community supervision status. There were no significant differences by community supervision status across seven indicators of healthcare confidence (*p*s > .05). However, results revealed significant differences in past experiences and beliefs about healthcare, health information seeking, and healthcare setting preferences by community supervision status (*p*s < .05), where women on community supervision less frequently sought health information and medical care outside of emergency departments.

**Conclusions:**

Findings provide preliminary evidence about differences in the healthcare experiences and setting preferences of women who inject drugs on community supervision.

Community supervision, defined as being on probation or parole, is significantly associated with greater physical, mental health, and substance use healthcare needs (Dong, Must, Tang, Beckwith, & Stopka, 2018). Yet this population is less likely to use outpatient healthcare services than other adults (Hawks et al., 2020). While legislation, such as the Affordable Care Act, has been enacted to increase insurance coverage among individuals recently released from incarceration or who are on community supervision (Bandara et al., 2015; Cuellar & Cheema, 2012; Knapp et al., 2019), improved insurance coverage has not sufficiently addressed gaps in outpatient service utilization rates among justice-involved individuals who have re-entered into the community and among the broader population of individuals on community supervision (Hawks et al., 2020; Saloner, Bandara, McGinty, & Barry, 2016). The continued underutilization of outpatient care among Medicaid enrollees has highlighted additional barriers to service use, such as perceived lack of coverage or unaffordable care, undervaluing primary care, length of time to first available appointment, and transportation (Saluja et al., 2019).

While the proportion of US adults under community supervision has steadily declined for a decade since 2008, Pennsylvania continues to exceed the national average by 64% as of 2018 (Kaeble & Alper, 2020). In Philadelphia, women experience disproportionately worse health outcomes after release from prison than men, including those on community supervision. In an analysis of Philadelphia state prison releases from 2010 to 2016, researchers found that women experienced significantly higher rates of all-cause and overdose-specific mortality after release compared to both their male counterparts and non-incarcerated adults (Pizzicato, Drake, Domer-Shank, Johnson, & Viner, 2018). Women who had been on probation or parole in the past year also have significantly greater odds of injection drug use compared to women with no justice involvement, even after controlling for housing stability (Lorvick, Comfort, Krebs, & Kral, 2015). Thus, the cross-section of women who are both justice-involved and who inject drugs is an important group of focus.

One way of explaining gender disparities in health outcomes and healthcare utilization rates among community supervised populations is through the lens of cumulative disadvantage. Women on community supervision experience multiple levels of adverse circumstances and opportunity gaps that coalesce into significant, cumulative social disadvantages relative to men (Bohmert & DeMaris, 2018), such as having significantly greater risk of experiencing intimate partner violence, having co-occurring mental health disorders, being unable to find employment, and having a history of trauma (Salem, Nyamathi, Idemundia, Slaughter, & Ames, 2013). Programs for women with co-occurring trauma and substance use disorders, such as integrated cognitive behavioral therapy programs implemented within carceral facilities (Zlotnick, Najavits, Rohsenow, & Johnson, 2003) and community settings (Hien et al., 2020), address the complex traumas experienced by justice-involved women with substance use disorders. The effectiveness of these integrated cognitive behavioral programs aligns with a recent meta-analysis that found preliminary evidence favoring integrated cognitive behavioral interventions for addressing co-occurring mental health and substance use over single-disorder interventions, such as interventions focused solely on either substance use or mental health (Mehta et al., 2021).

Stigma also explains gender disparities in healthcare experienced by women on community supervision, particularly among women who inject drugs (WWID). Stigma impacts healthcare access and utilization among women from socially marginalized groups, including justice-involved women (Salem et al., 2013; van Olphen, Eliason, Freudenberg, & Barnes, 2009) or who use alcohol and other drugs (Fraser, Moore, Farrugia, Edwards, & Madden, 2020; van Olphen et al., 2009). Among people who inject drugs (PWID), stigma adversely affects the patient-provider relationship and healthcare utilization through repeated processes of mistreatment and anticipation of subsequent mistreatment by providers and office staff (Biancarelli et al., 2019).

Given that women on community supervision and WWID have significant unmet health needs (Dion et al., 2020; Hawks et al., 2020) and experience significant social vulnerability (Lorvick et al., 2015), understanding how the combined experiences of community supervision and injection drug use influence women’s healthcare experiences and service setting preferences can inform how best to intervene with this population. To identify routes for addressing unmet healthcare needs, the present study examined how beliefs about healthcare, health information seeking behaviors, and healthcare setting preferences varied by community supervision status among a sample of WWID in Philadelphia.

## Methods

### Design

The study is a secondary analysis of a previously collected, cross-sectional survey that was conducted among a sample of one hundred women at risk for HIV who were actively utilizing a syringe exchange program in Philadelphia. The purpose of the original study was to develop targeted communication about pre-exposure prophylaxis (PrEP) for an intervention to promote uptake and adherence among WWID. The study was reviewed and approved by the Temple University Institutional Review Board, and all participants provided informed consent.

### Sample

Women were recruited from a syringe exchange and social service organization that provides services to people with substance use disorder in Philadelphia. Inclusion criteria included being self-reported female and at risk for HIV. Only those who reported current use of injection drugs were used in this analysis. Eighty-nine percent of the 64 eligible women reported currently taking opioids. Most women were White (75%), 9.4% were Black or African American, 7.8% were Hispanic or Latinx, and 7.8% identified as multiple races or another race. Women ranged in age from 23 to 54 years, with a mean age of 39 years (*sd* = 8.0). Health insurance was prevalent, with 86% reporting active coverage. Most women had completed high school or a GED (81%); 25% reported completing a college degree. Almost all (97%) had a history of incarceration.

### Measures

#### Individual characteristics

Self-reported individual characteristics included age, race and ethnicity, housing stability, educational attainment, monthly income in dollars, incarceration history, and current use of opioids.

#### Community supervision status

The independent variable was community supervision status, which was self-reported and operationally defined as currently being on probation or parole.

#### Healthcare confidence, healthcare beliefs, and health information seeking

Dependent variables included healthcare confidence, healthcare beliefs, and health information seeking. Using selected items from an adapted version of the Patient Self Advocacy Scale (Brashers, Haas, & Neidig, 1999), healthcare confidence was assessed with five questions and health information seeking was assessed with a single question. Healthcare beliefs were assessed with seven survey items developed from previous formative research. All items were assessed on a bipolar Likert scale, ranging from 0 to 10. Items were dichotomized as agreements vs. neutral or disagreements. The cut-point for defining agreement was a score of a six or higher, and a neutral or disagreement included ratings of five or below.

#### Healthcare access

Two binary survey items assessed healthcare access: 1) current insurance status and, 2) whether distance from healthcare facilities was a barrier to healthcare.

#### Healthcare setting preferences

Participants selected all of the healthcare settings at which they would prefer to receive healthcare services, in the event that they needed to receive medical care for themselves. Respondents were also given an option for preferring not to receive care. A binary variable was computed to identify individuals who preferred to only visit the emergency room or to not receive care. The remaining respondents selected at least one outpatient-, clinic-, or other community organization-based setting for receiving care.

### Analysis

Independent samples *t*-tests compared respondent mean age by community supervision status. Chi-square tests of independence identified significant differences in categorical individual characteristics and other study variables by community supervision status. If at least one cell had an expected count of fewer than five, significance was calculated using Fisher’s exact, two-sided test. All data were analyzed in IBM SPSS for Mac, Version 26.0.

## Results

Independent samples *t*-tests and chi-square tests revealed no significant differences on any individual characteristics by community supervision status. Results are reported in Table 1.

**Table 1:** Characteristics of WWID by community supervision status (*N* = 64)

	Not currently on probation or parole (*n* = 46)		Currently on probation or parole (*n* = 18)			
Variable	%	n	%	n	*X*^2^	*p*
Age, in years, *M* (*sd)*	38.8	(8.4)	39.6	(7.0)	0.381^a^	0.704
Race and ethnicity						
White, non-Hispanic	71.7%	33	83.3%	15	0.928	0.522^b^
Hispanic, Latinx, or not White	28.3%	13	16.7%	3		
Housing stability, past 6 months						
Has been homeless	91.3%	42	77.8%	14	2.164	0.206^b^
Has not been homeless	8.7%	4	22.2%	4		
Educational attainment						
HS graduate, GED, or less	58.7%	27	44.4%	8	1.060	0.303
At least some college	41.3%	19	55.6%	10		
Monthly income						
Less than or equal to $500/month	23.9%	11	38.9%	7	1.435	0.354
Greater than $500/month	76.1%	35	61.1%	11		
Incarceration history						
Has been in jail or prison	95.7%	44	100.0%	18	0.808	1.000^b^
Never been in jail or prison	4.3%	2	0.0%	0		
Current opioid use						
Yes	84.8%	39	100.0%	18	3.076	0.177^b^
No	15.2%	7	0.0%	0		

*Notes. WWID* Women who inject drugs, *HS* high school, *t*-*test* independent samples *t*-test, *X*^2^ chi-square test

^a^ Statistical significance was calculated using an independent samples *t*-test for age because it was a continuous variable

^b^ Statistical significance was calculated using Fisher’s exact, two-sided test because at least one cell had an expected count of fewer than 5

* *p* < .05

Chi-squared tests revealed no significant differences in healthcare access by community supervision status (*p*s > .05). There were also no significant differences by community supervision status across all seven indicators of healthcare confidence (*p*s > .05). However, significant differences in healthcare beliefs, health information seeking, and healthcare setting preferences emerged by community supervision status. For example, more WWID on community supervision reported that the emergency room was the easiest setting to receive medical care, *X*^2^ (1, *N* = 64) = 6.705, *p* = .01. Being on probation or parole was also associated with a preference for solely receiving care at only the emergency room or for receiving no medical care *X*^2^ (1, *N* = 64) = 4.187, *p* < .05. Finally, fewer WWID on community supervision reported actively seeking health information for themselves *X*^2^ (1, *N* = 64) = 5.957, *p* < .05. Results are reported in greater detail in Table 2.

**Table 2:** Healthcare confidence, past experiences and beliefs about healthcare, healthcare access, health information seeking, and healthcare setting preferences by community supervision status among WWID (*N* = 64)

	Not currently on probation or parole (*n* = 46)		Currently on probation or parole (*n* = 18)		*X*^2^	*p*
Variable	%	n	%	n		
*Healthcare confidence*						
Lack of confidence negatively affects healthcare	21.7%	10	44.4%	8	3.299	0.069
Feels more assertive than other WWID	56.5%	26	50.0%	9	0.222	0.637
Asks doctor questions about prescriptions	87.0%	40	83.3%	15	0.141	0.703^a^
Will not do treatment if disagrees with it	73.9%	34	77.8%	14	0.103	1.000^a^
Don’t always follow doctor’s instructions	63.0%	29	61.1%	11	0.021	0.886
*Past experiences and feelings towards healthcare*						
Feels judged by doctor and doctor’s office	26.1%	12	27.8%	5	0.019	1.000^a^
Feels that doctors don’t want to treat people like them	23.9%	11	33.3%	6	0.589	0.533^a^
Experiences positive interactions with doctor office staff	71.7%	33	72.2%	13	0.001	0.969
Feels comfortable talking with doctors	71.7%	33	72.0%	13	0.001	0.969
Feels doctors listen to them and do not rush them	65.2%	30	66.7%	12	0.012	0.913
Experiences ease with obtaining doctor appointment	45.7%	21	77.8%	14	5.388	0.020*
Feels ER is easiest for medical care	54.3%	25	88.9%	16	6.705	0.010*
*Healthcare access*						
Has health insurance	84.8%	39	88.9%	16	0.181	1.000^a^
Distance is a barrier for getting to doctor appointment	30.4%	14	33.3%	6	0.051	0.822
*Health information seeking*						
Actively seeks health information for self	71.7%	33	38.9%	7	5.957	0.015*
*Healthcare setting preferences*						
Prefers to use ER only or does not use medical care	28.3%	13	55.6%	10	4.187	0.041*

*Notes*. *WWID* women who inject drugs, *ER* emergency room, *X*^2^ chi-square test

^a^ Statistical significance was calculated using Fisher’s exact, two-sided test because at least one cell had an expected count of fewer than 5

* *p* < .05

## Discussion

The current study presents preliminary evidence towards differences in specific healthcare experiences and preferences of women on community supervision who are actively using injection drugs. These results align with the current literature which cites social vulnerability (Lorvick et al., 2015) and cumulative social disadvantage (Bohmert & DeMaris, 2018) as determinants of decreased healthcare utilization among WWID and women on community supervision.

The current study found that being on probation and parole was significantly associated with a sole preference for using the emergency room for care or for not receiving any care. A stated preference for the emergency department is noteworthy, given that using the emergency room for usual care is associated with worse patient care experiences, less receipt of high-value clinical care (Levine, Landon, & Linder, 2019), and less continuity of care (Pourat, Davis, Chen, Vrungos, & Kominski, 2015). Among PWID, mental health is the leading reason for emergency room visits (Dion et al., 2020), yet the emergency room mental health treatment is fraught with challenges that result in negative patient experiences, such as a lack of dignity, long wait times, and decreased autonomy (Thomas et al., 2018). Preference for emergency services may result from avoiding preventative and non-emergency medical care, strategies commonly used by PWID to limit exposure to the injection drug use stigma that remains prevalent in healthcare settings (Biancarelli et al., 2019). Primary care services may also be deprioritized by women on community supervision due to higher ranking daily needs, such as food and housing security (Dong et al., 2018), resulting in greater use of acute care services. In addition, a preference for emergency departments over other healthcare settings aligns with recent qualitative findings that Medicaid and public insurance enrollees described emergency departments and urgent care clinics as faster, cheaper, and more familiar than outpatient primary care settings (Saluja et al., 2019).

Finally, it is significant that these women were significantly less likely to search for health information for themselves. The intersectional stigma of being on community supervision, being female, and using injection drugs may result in higher stress and a less supportive social environment, which are factors associated with lower health literacy and patient engagement (McCormack, Thomas, Lewis, & Rudd, 2017). Further investigation is needed into the specific barriers, including multilevel stigma, that inhibit active health information seeking and outpatient healthcare use by WWID on community supervision to better address their unmet health needs. The capacity for community corrections officers to improve and help foster their clients’ active health information seeking and health literacy skills should also be explored.

Previous programs that have been implemented to increase outpatient and primary care use among individuals recently released from incarceration have included integrated and specialized primary care services for justice-involved individuals (Howell et al., 2021; Morse et al., 2017; Wang et al., 2019), patient navigation (e.g., Binswanger, Whitley, Haffey, Mueller, & Min, 2015; O’Connell, Visher, & Becker, 2020), and systems change interventions (O’Connell et al., 2020). In a study of women released from incarceration who were referred by community health workers to Transitions Clinic Network, a specialized integrated primary care clinic for justice-involved individuals, about one half of participants were successfully linked to primary care (Morse et al., 2017). Qualitative analyses among a subgroup of program participants indicated that women felt increased empowerment, autonomy, health literacy, and competence to address health conditions (Thomas, Wilson, Bedell, & Morse, 2019). At another site within the Transitions Clinic Network, researchers found that the provision of specialized primary care services for formerly incarcerated individuals was also associated with lower recidivism rates (Wang et al., 2019). While the Transitions Clinic Network is a national network, Pennsylvania does not have any sites as of March 2021 (Transitions clinic network sites, n.d.).

In a recent trial, patient navigation was associated with a significantly greater percentage of individuals on probation receiving referrals and linkage to primary care compared to control group participants who only received informational booklets (O’Connell et al., 2020). Notably, this pilot by O’Connell et al. (2020) concurrently used a system change intervention which used local change teams to more effectively facilitate linkage to primary care through intersectoral collaborations (e.g., community corrections agencies and officers, clients, and healthcare providers), an approach that has been used to connect justice-involved individuals with HIV testing (Belenko et al., 2017) and substance use disorder treatment (Elkington, Lee, Brooks, Watkins, & Wasserman, 2020; Howell et al., 2021; Martin et al., 2021; McCarty et al., 2007). Support for these approaches is encouraging (Belenko et al., 2017), but several of these studies are still ongoing (e.g., Howell et al., 2021; Martin et al., 2021). It should also be noted, however, that the syringe exchange that women in this study used also has significant healthcare resources available to participants, yet the difference in preference for healthcare location was still found. Thus, gender-specific outcome data are needed to better understand the mechanisms by which specialized integrated primary care clinics, patient navigation, and systems change interventions could help to address injection drug use and justice system stigma, social vulnerability, and the healthcare needs of women who inject drugs on community supervision.

### Limitations

The results of this study should be interpreted with the recognition of several key limitations. For example, injection drug use stigma alone may already account for a significant portion of the variation in the healthcare experiences of women. Over one quarter of respondents felt that doctors judged them and did not want to treat people like them. Small sample size also limits external validity and the possibility of using some types of statistical analyses, and cross-sectional data limits potential for causal inferences. Finally, drug use stigma and the experiences of individuals on community supervision vary regionally, so this sample of Philadelphian women may not generalize to all US adults.

## Conclusion

There is significant underutilization of healthcare in outpatient and clinic-based settings by WWID on community supervision, and initiatives to increase healthcare access have not addressed this gap. To better meet the healthcare needs of WWID on community supervision, it is necessary to look beyond insurance coverage to identify targeted methods of increasing interest in and engagement with outpatient, clinic-based, and community organization-based healthcare providers. More data, particularly gender-specific data, are needed to explore the utility of integrated and intersectoral approaches for addressing the complex healthcare needs and underutilization of outpatient care settings among women who inject drugs on community supervision.

## Data Availability

The dataset analyzed during the current study is available from the corresponding author on reasonable request.
